# Success in Competition for Space in Two Invasive Coral Species in the western Atlantic – *Tubastraea micranthus* and *T*. *coccinea*

**DOI:** 10.1371/journal.pone.0144581

**Published:** 2015-12-18

**Authors:** Paul W. Sammarco, Scott A. Porter, Melissa Genazzio, James Sinclair

**Affiliations:** 1 Louisiana Universities Marine Consortium (LUMCON), 8124 Hwy. 56, Chauvin, LA 70344-2110, United States of America; 2 Department of Oceanography and Coastal Sciences, Louisiana State University, Baton Rouge, LA 70803, United States of America; 3 EcoLogic Environmental, Inc., PO Box 886, Houma, LA 70361, United States of America; 4 Center for Marine Science, University of North Carolina at Wilmington, 5600 Marvin Moss Ln., Wilmington, NC 28409, United States of America; 5 US Department of the Interior, Bureau of Safety and Environmental Enforcement, 1201 Elmwood Park Blvd., New Orleans, LA 70123-2394, United States of America; National Taiwan Ocean University, TAIWAN

## Abstract

Invasion success by an alien species is dependent upon rate of reproduction, growth, mortality, physical characteristics of the environment, and successful competition for resources with native species. For sessile, epibenthic marine species, one critical resource is space. We examined competitive success in two invasive Indo-Pacific corals involved in competition for space in the northern Gulf of Mexico—*Tubastraea coccinea and T*. *micranthus*—on up to 13 offshore oil/gas platforms south of the Mississippi River. Still-capture photos of thousands of overgrowth interactions between the target corals and other sessile epibenthic fauna were analyzed from ROV videos collected at 8–183 m depth. *T*. *micranthus* was observed overgrowing >90% of all sessile epibenthic species which it encountered. Frequencies of competitive success varied significantly between platforms. *T*. *coccinea* was competitively superior to all competitors pooled, at the 60% level. There was little variability between *T*. *coccinea* populations. *T*. *coccinea* encountered the following species most frequently—the encrusting sponges *Xestospongia* sp. (with the commensal *Parazoanthus catenularis)*, *X*. *carbonaria*, *Dictyonella funicularis*, *Mycale carmigropila*, *Phorbas amaranthus*, and *Haliclona vansoesti*—and was found to be, on average, competitively superior to them. Both *T*. *micranthus* and *T*. *coccinea* appear to be good competitors for space against these species in the northern Gulf of Mexico. Competitive success in *T*. *micranthus* was highest in the NE part of the study area, and lowest in the SW area near the Mississippi River plume. *T*. *coccinea*’s competitive success peaked in the SW study area. This suggests that variation in competitive success both within and between populations of these species may be due to differences in local environmental factors.

## Introduction

Many non-native species have been regarded as invasives because of the problems they have caused for the communities they have colonized [1]. Data regarding the impacts of different invasive species are disparate and sometimes equivocal [2]. In some cases, they have displaced some native species entirely [3]. In others, they have become dominant in the community [4]. In still others, they have adapted to their new environments, becoming an integral part of the community without causing local extinction or major changes to the community [5].

Over the past 70 yrs, there have been several invasions of coral species to the western Atlantic. One was that of *Fungia scutaria* [6,7,8], which was introduced to northern Jamaican waters in Discovery Bay in the 1960s/1970s. *Fungia* is a vagile Indo-Pacific fungiid coral and lives primarily on soft-bottom [9]. It has not yet been reported to have any major effect on the benthic community and appears to have integrated well into the community. It probably invaded an open niche in its Caribbean soft-bottom environment (see [10] for description of unique feeding habits). A second suite of corals also invaded the western Atlantic, all species of which belonged to the genus *Tubastraea*. Firstly, the Indo-Pacific species *T*. *coccinea* was recorded in Puerto Rico during the 1940s, most likely having been carried through the Panama Canal on the hull of a ship [11]. It rapidly spread to the Netherlands Antilles and, from there, points in all directions, as far south as Brazil and as far north as the Gulf of Mexico and the Florida Keys [12,13,14,15,16,17,18,19]. This geographic range expansion required 60–70 yrs. More recently, *T*. *tagusensis* and *T*. *coccinea* have been reported to have invaded the Tamoios Ecological Station Marine Protected Area (MPA) in Brazilian waters, with rapidly expanding populations [20].

We recently reported that *Tubastraea micranthus* invaded an area south of the Mississippi River mouth [21], having colonized offshore oil and gas platforms in this area. Later, we found that their populations were spreading rapidly throughout this region [8]. They have been found to extend to depths below the depth-distribution of the co-occurring congener *T*. *coccinea* [22]. *T*. *coccinea* extends to depths of ~50 m, while *T*. *micranthus* can extend to depths of at least 183 m.

Knowledge of population growth rates, rates of range expansion, and depth distributions, along with rates of colony growth, reproduction, or mortality [23] are important in attempting to predict the potential success of a biological aquatic invasion [24,25,26]. Another important factor influencing invasion success is the ability of the invading species to out-compete native species for local resources [3,27,28]. In the case of a benthic or demersal species, and particularly for sessile epibenthic species, this means competition for space [29,30,31]; also see [32], which is critical to survival. If a species cannot compete successfully for space, it will not succeed in its invasion, despite high potential rates of colony growth, reproduction, larval dispersal, etc. [23,33]. Certainly, local environmental variables also influence the ability of a new foreign species to survive—including temperature and salinity [34], nutrient concentrations [35,36], and turbidity, sedimentation, and light [37] and potential exposure to native diseases. All of these factors can contribute to the success or failure of a species introduction.

One of the most effective means that some groups of cnidarian marine organisms have of expanding their territory is through extracoelenteric digestion [38,39,40] or the development of sweeper tentacles [41]. Scleractinian corals in particular use this mechanism to defend and acquire space from neighboring sessile epibenthic fauna; it can be a very effective means of gaining and maintaining living space in invasive species. Mesenterial filaments are extruded from the gut of the coral, and extend some distance to touch the neighbor. Batteries of nematocysts housed in the filaments are discharged on the neighbor, resulting in death of that portion of the colony (see [31] for review). Other types of interactions occur as well. A common set of interactions may be found on coral reefs occurring between corals and sponges competing for space [31,42,43,44,45,46]. One such interaction is allelopathy, which has been demonstrated in numerous terrestrial plants [47,48] and Indo-Pacific alcyonacean soft corals on the Great Barrier Reef [49,50,51,52]. Another type of interaction is direct overgrowth of one species by another [53,54], as in *Millepora alcicornis* overgrowing *Gorgonia ventalina* [55]. Another is biological disturbance whereby locally generated currents are used to divert food away from another neighboring organism, as occurs between bryozoans, sponges, and some other organisms [56,57].

Here, we have assessed the frequency of competitive success in the Indo-Pacific coral species *Tubastraea micranthus* against other sessile, epibenthic fauna occurring on pilings of a number of offshore oil and gas platforms in the northern Gulf of Mexico. We assess the frequency of overgrowth and frequency of being overgrown as an indicator of success in competition for space in this new invasive species with respect to other species it encounters during colony growth. We also report the same information for *T*. *coccinea*, a congeneric invasive species which colonized the Caribbean over 70 yrs ago. We are considering the competitive abilities of these two invasive scleractinian species as potential characters which could contribute to their success as invasive species in the Gulf of Mexico and other points in the Caribbean.

## Materials and Methods

### Study site

**Fig 1:**
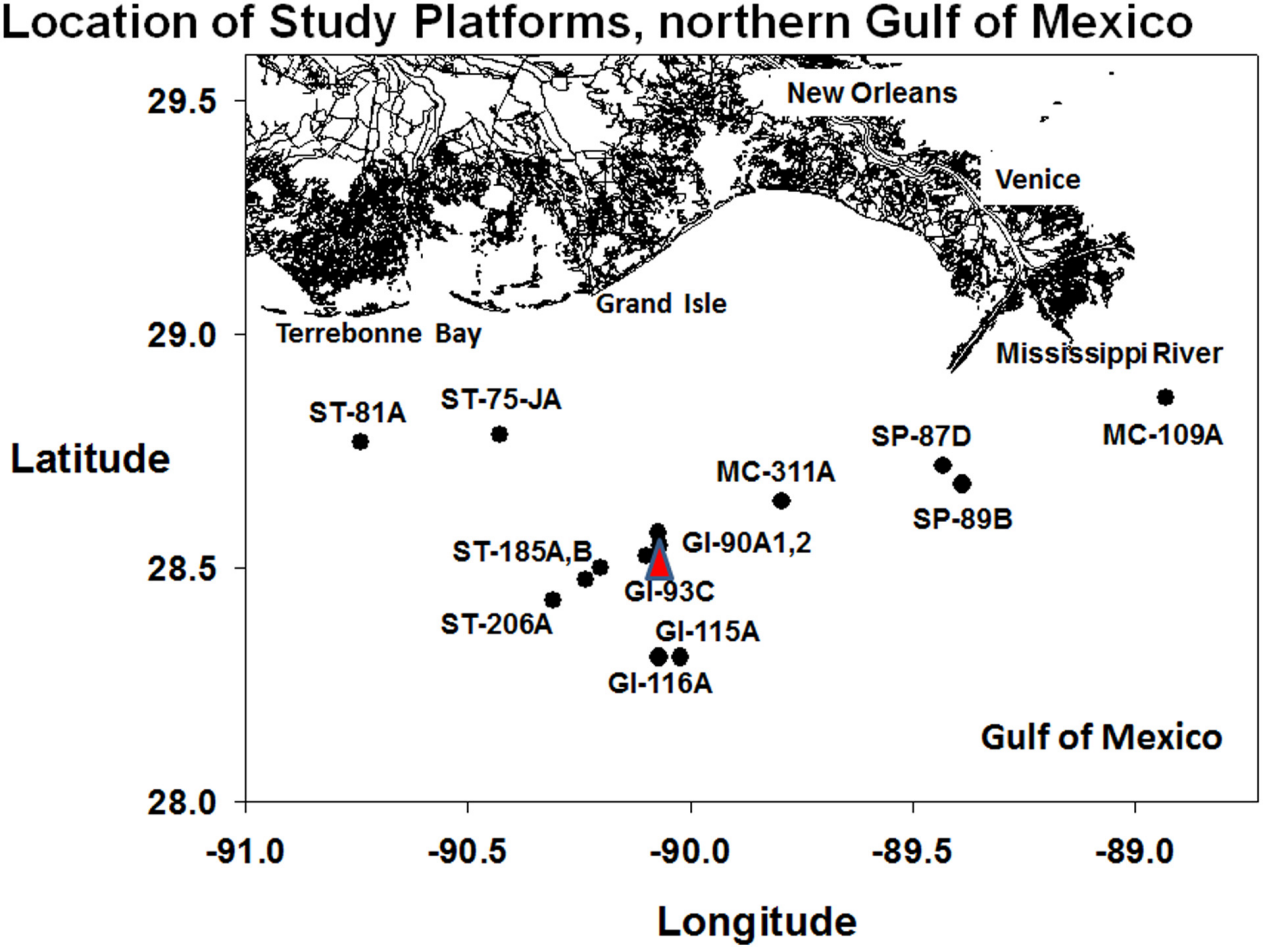
A map of all platforms surveyed by ROV during this study. Platform code and position shown. Point of first sighting (2007) shown as a triangle (GI-93C).

A total of fifteen platforms within a 25 km radius south of the mouth of the Mississippi River were initially surveyed (Fig 1; Table 1; [8]. Nine platforms possessed *Tubastraea micranthus* in sufficient numbers to be statistically analysed for frequency of competitive success. *T*. *coccinea* was much more abundant, occurring in high densities on 13 platforms, and was assessed for frequency of competitive success on them. The platforms were chosen because of their proximity to the original sighting area of *Tubastraea micranthus*, and their accessibility for research purposes. They were not chosen in relation to any geographical pattern of placement; in that sense, the geographic distribution of the platforms within the study area was random. The M/V *Fling* (33 m, Fling Charters, Inc., Freeport, Texas) was chartered to support field operations, as was the R/V *Acadiana* (18 m, Louisiana Universities Marine Consortium—LUMCON). Remotely operated vehicle (ROV) surveys and laboratory analyses were conducted over a two-year period, using 12 days of ship-time. Two-thirds of one day were required to complete an ROV survey on a single platform.

**Table 1:** A list of all platforms surveyed by ROV during this study.

Platform			
Code	Owner	Latitude	Longitude
**GI-90A-1**	Apache Corp., Lafayette, LA	28.575144	-90.072429
**GI-90A-2**	Apache Corp.	28.575144	-90.072429
**GI-93C**	Apache Corp.	28.548886	-90.068677
**GI-94B**	Apache Corp.	28.540000	-90.275000
**GI-115A**	Walter Oil & Gas Corporation, Houston, TX	28.3076123	-90.0219665
**GI-116A**	Apache Corp.	28.30928306	-90.07054334
**MC-109A**	Stone Energy Corporation, Lafayette, LA	28.86467752	-88.93079054
**MC-311A**	Apache Corp.	28.642636	-89.794241
**SP-87D**	Apache Corp.	28.72001853	-89.43078669
**SP-89B**	Apache Corp.	28.680464	-89.387596
**ST-75-JA(B)**	Stone Energy Corporation	28.76955709	-90.74085664
**ST-81A**	Stone Energy Corporation	28.78656092	-90.42747823
**ST-185A**	Black Elk Energy Offshore Operations, LLC Houston, TX	28.495501	-90.203098
**ST-185B**	Black Elk Energy Offshore Operations, LLC	28.47493	-90.235942
**ST-206A**	Apache Corp.	28.45372522	-90.38341283

Platform code, oil or gas company owner, and geospatial position via latitude and longitude are given above. GI = Grand Isle lease sector; MC = Mississippi Canyon; SP = South Pass; ST = South Timbalier.

### Survey technique

No specific government or regulatory permits or permissions were required to perform this study. As a courtesy, however, permission to conduct surveys on the offshore platforms were obtained from the oil/gas companies which owned the platforms concerned, prior to sampling. These companies are listed in Table 1. For those interested in conducting future studies, the production supervisor or legal section of the oil/gas company in question can be contacted to obtain permission. No collections of any benthic organisms, including corals or any vertebrates associated with the sites, were made here. Only videographic data were collected via a ROV.

A Deep Ocean Engineering Phantom S2 ROV was used to conduct the surveys. We employed techniques used in earlier surveys [8,17,21,22]. Two back-up units were used—two SeaBotix LBV-300s, owned and operated by ARACAR (Alliance for Robot-Assisted Crisis Assessment) and BOEM (Bureau of Ocean Energy Management), respectively. All units possessed vertical and horizontal propulsion units, site-to-surface color video transmission, a monitor, forward-directed lights, reference laser beams with a known inter-beam distance, and fixed-grab capabilities for sample retrieval.

Surveys extended from 8 m to 138 m depth (the maximum depth of our deepest platform). The down-current side of the platform was always surveyed. This was done in order to keep the ROV as stable as possible (maneuvering into the current) during the video survey. It was also critical to keep the ROV umbilical from being drawn into the interior of the jacket by local currents and becoming entangled there. Currents in this region are not constant and can readily shift direction. Thus, the down-current side of the platform at our time of sampling did not necessarily mean we were only sampling the environment on one side of the platform.

On each platform, vertical pilings were surveyed as well as two sets of horizontal support struts, usually at depths of 13–17 m, and 21–27 m. The number of vertical pilings surveyed on each platform (2–4) depended upon the number of pilings available for survey as well as wind and sea conditions at the time of sampling. This affected sample size/number of quadrats analysed per platform. Data, however, were treated as proportions or frequencies. Any changes in sample size between platforms would have affected the power of the test [66] for that specific comparison. If any bias was introduced in this way, it would have made the statistical tests more conservative.

Video and still images were processed using a Dell Precision 340, a T3400 desktop computer, a Dell Precision M4300 Workstation, and MicroSoft video imaging software. We also used Nero 7.0, VideoLAN, and MicroSoft Windows Media Player, which were capable of zoom and still-image capture. Still images approximately 15 x 15 cm in screen-size were frozen and analyzed. One quadrat was analyzed for every 2 m of depth per transect on vertical pilings. Up to four replicates per depth per platform were captured and analyzed for both *Tubastraea micranthus* and *T*. *coccinea*. A transparent 25.4 x 25.4 cm grid, split into squares 2.54 x 2.54 cm in size, was placed in the center of the computer screen and used as a reference guide to facilitate sampling competitive interactions and data collection. Because of potential movement of the ROV while maneuvering into the current, it was difficult to maintain a position precisely at a given distance away from the pilings. It was possible, however, to calibrate image sizes within the still frames and standardize the sampling by using two reference lasers, 10 cm apart and clearly visible within the view. The laser beams were transmitted by the ROV horizontally onto the substrate.

In order to make comparisons regarding the competitive success frequencies of these two congeneric invasive coral species, data were collected for both *Tubastraea micranthus* and *T*. *coccinea*. *T*. *coccinea* population densities were extraordinarily high [17]; thus, in that case, sample size regarding competitive interaction data was limited to five colonies per quadrat. Colonies were selected in a haphazard manner to avoid bias associated with colony selection. The small sample sizes of *T*. *coccinea* per quadrat were compensated-for by the large number of quadrats sampled per platform (> 1,000 quadrats/platform in some cases).

Evidence of inter-specific competition involving the co-occurring sessile epifauna was recorded. This included any intra- or interspecific competition between the two target species. The sessile epibenthic community comprising the surfaces of platform jackets has been described in detail by Gallaway and Lewbel [58], and Lewbel et al. [59]. At the time of the most recent survey [59], the community was found to be dominated by cirripedes (barnacles) and pelecypods (bivalves) in shallow water. Other abundant groups included actinarian and zoanthid anemones, bryozoans, caprellid and gammaridian amphipods, clionid sponges, hydroids, ophiuroids, and tunicates. Both sub-tropical and tropical coral reef species were found there. Algae were also prominent near the surface, particularly on near-shore platforms. Since that time, the platforms of the northern Gulf of Mexico have been colonized and dominated by *Tubastraea coccinea* [16,17], which has most likely changed the community composition, or at least the relative abundances, of these taxonomic groups.

The techniques used here to assess competition for space in these organisms were similar to those used previously by Sammarco [60,61] and Sammarco and Carleton [62]. Sessile epibenthic organisms occurring within 1 cm of the target coral were assessed for interaction; no direct interactions were observed between species at or beyond this distance. Species encountered in the study were identified via still-capture photographs from the video surveys using a variety of taxonomic guides [13,63,64]. Competitive encounters were judged from the target coral’s perspective. The categories for which data were collected were 1) overgrowth of competitor by the coral; 2) overgrowth of the coral by the competitor; 3) simultaneous overgrowth of coral and competitor by each other (possible through their colonial growth form); 4) abutment (contact but no interaction; a stand-off); and 5) association only (occurring in the same immediate area, with no contact and no interaction). An observation of direct overgrowth by one species over another was interpreted to be evidence of success in competition for space, as has been used in previous related studies [31,53,54]. Category 3 was counted as a win for both species. Categories 4 and 5 were not counted as competitive successes for either species. If overgrowth of tissues was not clear, the interaction was scored as abutment/no interaction and no scoring was recorded [60,61]. Intraspecific interactions within either target species were not observed. Competitive networks may exist in this system, but it was not the objective of this study to assess or document such [53,54]. Patchiness in the distribution of populations of target coral species between the platforms and variations in their abundances was not considered in assessment of competitive outcomes here. The outcomes were considered to be, for the most part, genetically and immunologically controlled at the micro-level with respect to a portion of a single colony.

It was not the objective of this study to assess mechanisms of overgrowth and competition for space; this has been reported elsewhere [65]. The focus here was on a quantitative approach to assessing frequency of competitive success between the corals and co-occurring sessile epibenthic species. The competition variable was defined simply as the number of competitive successes over the total number of interactions within that set of interactions. A frequency of overgrowth significantly greater than 50% within sampling of the benthic community on a given platform was considered to be an indicator of average competitive superiority of the target species over the associated species. No attempt was made to consider competition for space between other species of sessile epibenthic fauna and flora as a community; *i*.*e*., this was not a community competition study. Only averages based on percent wins against individual competitors within the community are presented.

Of the 15 platforms surveyed, only those platforms which possessed a sufficient number of given paired interactions received quantitative analysis and are shown in the figures. (For G-statistic-based frequency analyses, the minimum cell frequency should be 3–5; [66]). 95% confidence limits are also shown in figures and are based upon total number of interactions observed on a given platform, using Sokal and Rohlf’s [66] and Rohlf and Sokal’s [67] confidence limits for percentages. Sample size ranges are provided in figure legends.

Here we have assessed a snapshot in time of a set of dynamic interactions between organisms, quantifying them, and making statistical comparisons among competitively interacting organisms [31,32,35,42,43,44,46,49,53,54,56]. This approach can be helpful in indicating competitive capabilities in the study organisms and will facilitate our understanding of ecological interactions between these competitors for space. This we judged from the viewpoint of competitive success frequencies through the collection of data in a standardized and controlled manner. Thousands of interactions were assessed and analyzed. Different platforms had different sample sizes, due to different coral population sizes. Small sample sizes will, of course, affect the power of the test; those platforms falling into this category may have yielded non-significant results when, under conditions of higher sample sizes, they might otherwise have been significant [66].

Evidence of tissue damage or necrosis was not considered necessary to score overgrowth as competitive success. A variety of interactions were, however, observed in passing. For example, no white necrotic bands were observed in interactions between the corals and the sponges, although such was occasionally observed between sponges in contact. Direct overgrowth was commonly observed. Abutments or stand-offs were observed. Active release of nematocysts were not observed, nor were sweeper tentacles. Production of mesenterial filaments or the use of extracolenteric digestion was not observed, although this is now known to occur in both of these *Tubastraea* spp. [17,18,65]. This was a study extending into deep water, performed with the assistance of an ROV using video photography and single still-capture photographs at a point in time. Because of this, it was not possible to assess the effects of partial mortality in specific colonies through time, including effects of predation by other organisms, aging processes, other physical damage, etc. As noted earlier, it was not the objective of this study to study temporal changes in competition for space.

### Statistical Analysis

Statistical data analyses were performed using BiomStat 3.2 and 3.3 [68,69]. Data were pooled by platform to provide an overview of competitive interactions or percent competitive success. The term “different from”. “variation from”, etc. will be used wherever a statistical comparison has been found to be “significantly different” or “vary significantly from”. That is, the term “significant” will be implied.

The number of interactions per platform was ≤1,283. The data were tested for variation from a 1:1 ratio of competitive successes to losses. Fisher’s Exact Test was used to determine whether the frequency of competitive success on a given platform was greater than 50% for a given set of interactions. Differences in competitive success between platform populations were examined via a Goodness of Fit Test using the G-statistic, again, against a 1:1 expected ratio of win to loss [66]. Arcsine transformations were applied in graphic representations for percentages in those cases where values were below 30% or above 70%, for normalization purposes [66]. *A posteriori* comparisons were made between coral populations on the various platforms using Goodness of Fit tests (G-statistic) [66]. Data were plotted as bar diagrams using SigmaPlot 10.0. Details of statistical results are presented in the figure legends. Only statistically significant results will be discussed.

## Results

**Fig 2:**
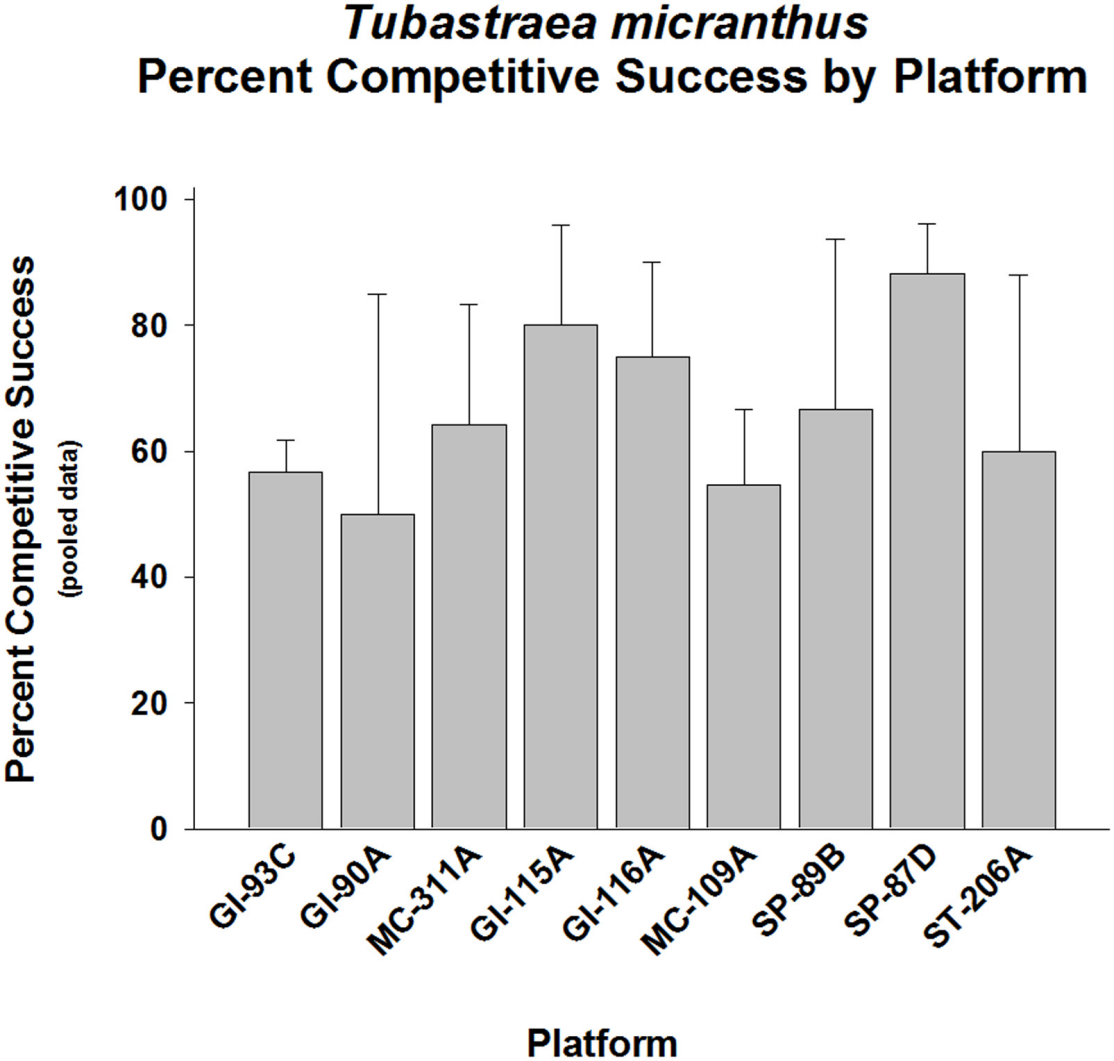
Percent wins in competition for space between *Tubastraea micranthus* and all other sessile epifauna pooled, by platform. Percent competitive success plus 95% confidence limits shown. The competition variable is defined as the number of competitive successes over the total number of interactions within a taxonomic group. An overgrowth frequency of significantly >50% was considered to be an indicator of competitive success. A total of 15 platforms were surveyed. Those platforms possessing a number of interactions sufficient for quantitative analysis were included in the study. Data tested for significant variation from 1:1 ratio of competitive successes to losses. Range of number of interactions per platform (n): 18–361. Overall competitive success was significantly higher than the expected 50% level over all platforms (p < 0.01, Fisher’s Exact Test). Highly variable win frequencies between platforms (p < 0.001, Goodness of Fit Test, G-statistic). No significant sub-sets of platforms.

When data were pooled for interactions between *Tubastraea micranthus* and its competitors, the ROV videos revealed that, overall, *T*. *micranthus* was a better competitor for space than its associates (p < 0.001, Goodness of Fit Test, G-statistic; Fig 2, S2 Table). The percent of competitive successes by *T*. *micranthus* over other epibenthic organisms ranged from ~50% on GI-90A to ~90% on Platform SP-87-D. The competitive success frequencies were highly variable between platforms, with about half of them being higher than 50% (p < 0.05–0.01) and half of them not (p>0.05). Variability in paired interactions was observed, which implies that competitive interactions between any two species were not absolute.

**Fig 3:**
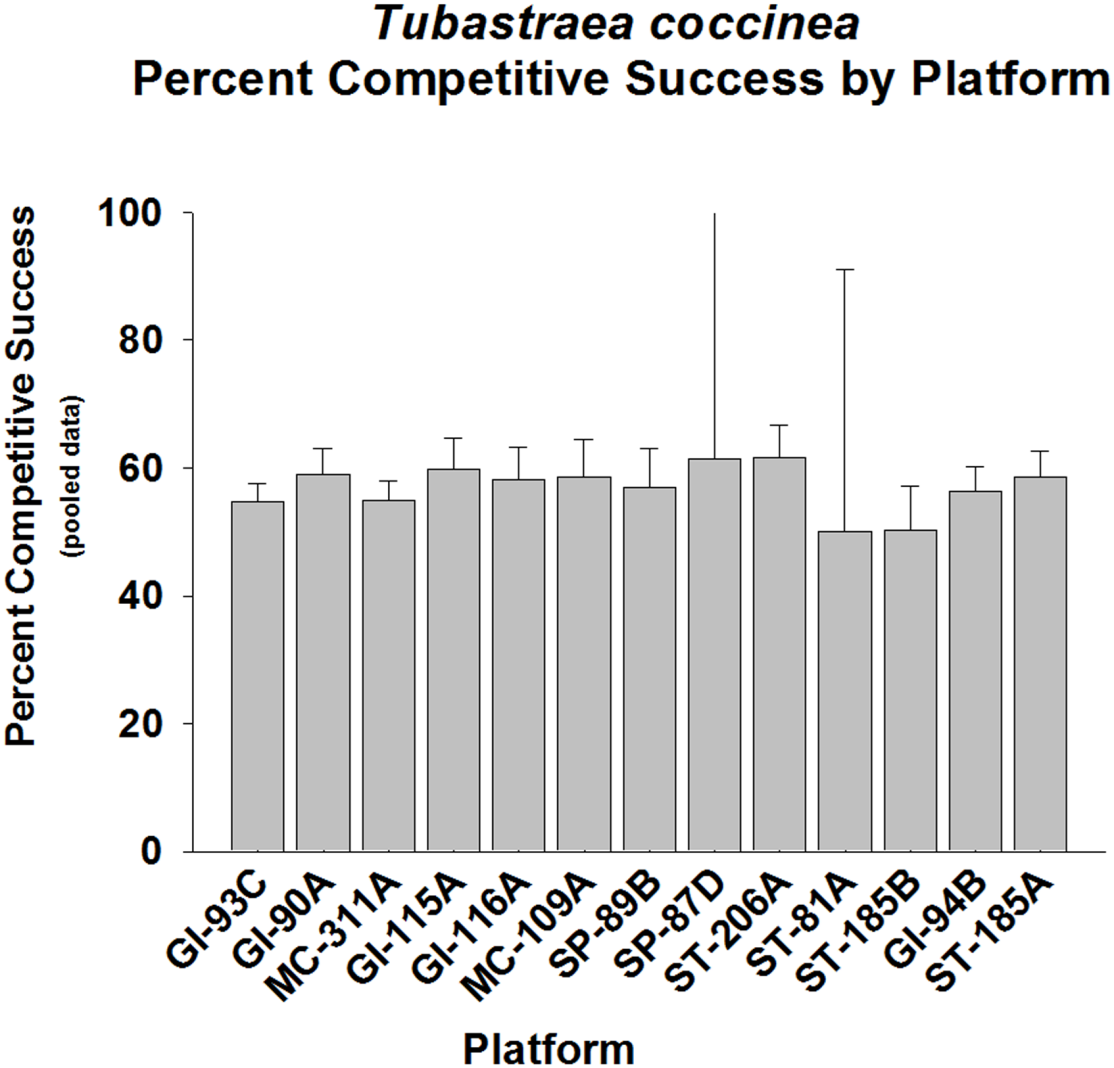
Percent success in competition for space between *Tubastraea coccinea* and all other sessile epifauna pooled, presented by platform. Mean plus 95% confidence limits shown. Range of number of interactions per platform (n): 217–1,283. See Fig 2 legend for additional details. Overall competitive success significantly higher than 50% (p < 0.001, Fisher’s Exact Test). Also, almost all platforms exhibited a competitive win frequency significantly higher than 50% (p < 0.01–0.001, Goodness of Fit Test, G-statistic), except ST-81A and ST-185B, which exhibited no significant difference from the 50% competitive success level (p > 0.05).

Analysis of competitive success in *Tubastraea coccinea* against all other sessile epibiota pooled revealed a strikingly uniform average of ~55% wins across most platforms, although some variation was also observed between platforms (p < 0.01, Fisher’s Exact Test; p < 0.01–0.001, Goodness of Fit Test, G-statistic; Fig 3, S2 Table). The only platform populations which exhibited a frequency of competitive success which was not different from 50% were ST-81A and ST-185B. The low variability observed in these interactions is indicative of a predictable ability of this species to compete successfully for space.

**Fig 4:**
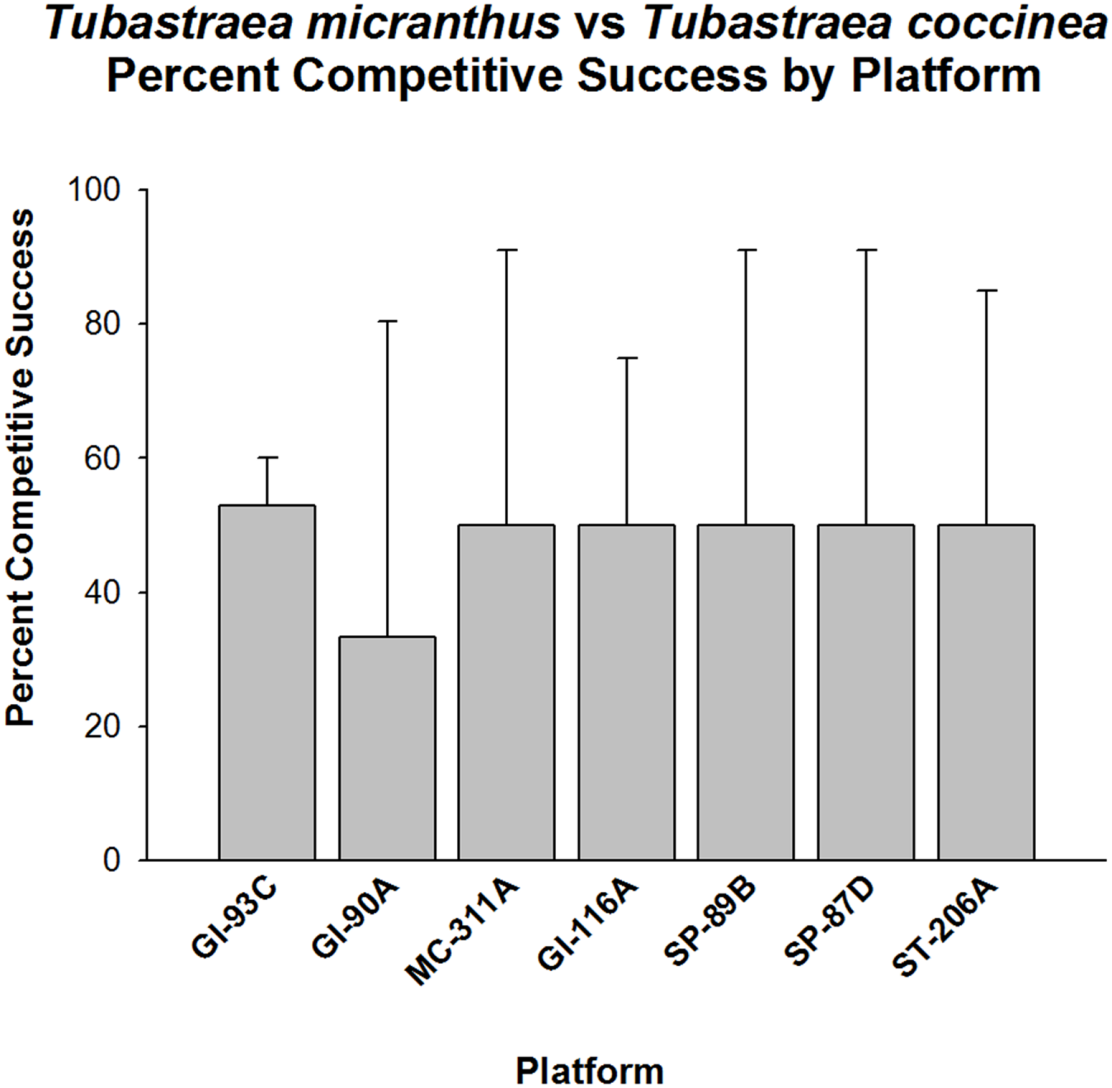
Percent success in competition for space between *Tubastraea micranthus* vs. *T*. *coccinea*. Here, the former species is the target organism. Presented by platform. Mean plus 95% confidence limits shown. See Fig 2 legend for additional details. No significant difference in overall competitive success (p > 0.05, Fisher’s Exact Test).

When frequency of competitive success was considered between the two target species—*Tubastraea micranthus* against *T*. *coccinea*—they were found to have similar competitive abilities (p > 0.05, Fisher’s Exact Test, Fig 4). This response did not appear to vary between platform populations, although small sample sizes may have obscured the trends observed here. The frequency of competitive success averaged ~50% on all platforms where they were in contact with each other.

**Fig 5:**
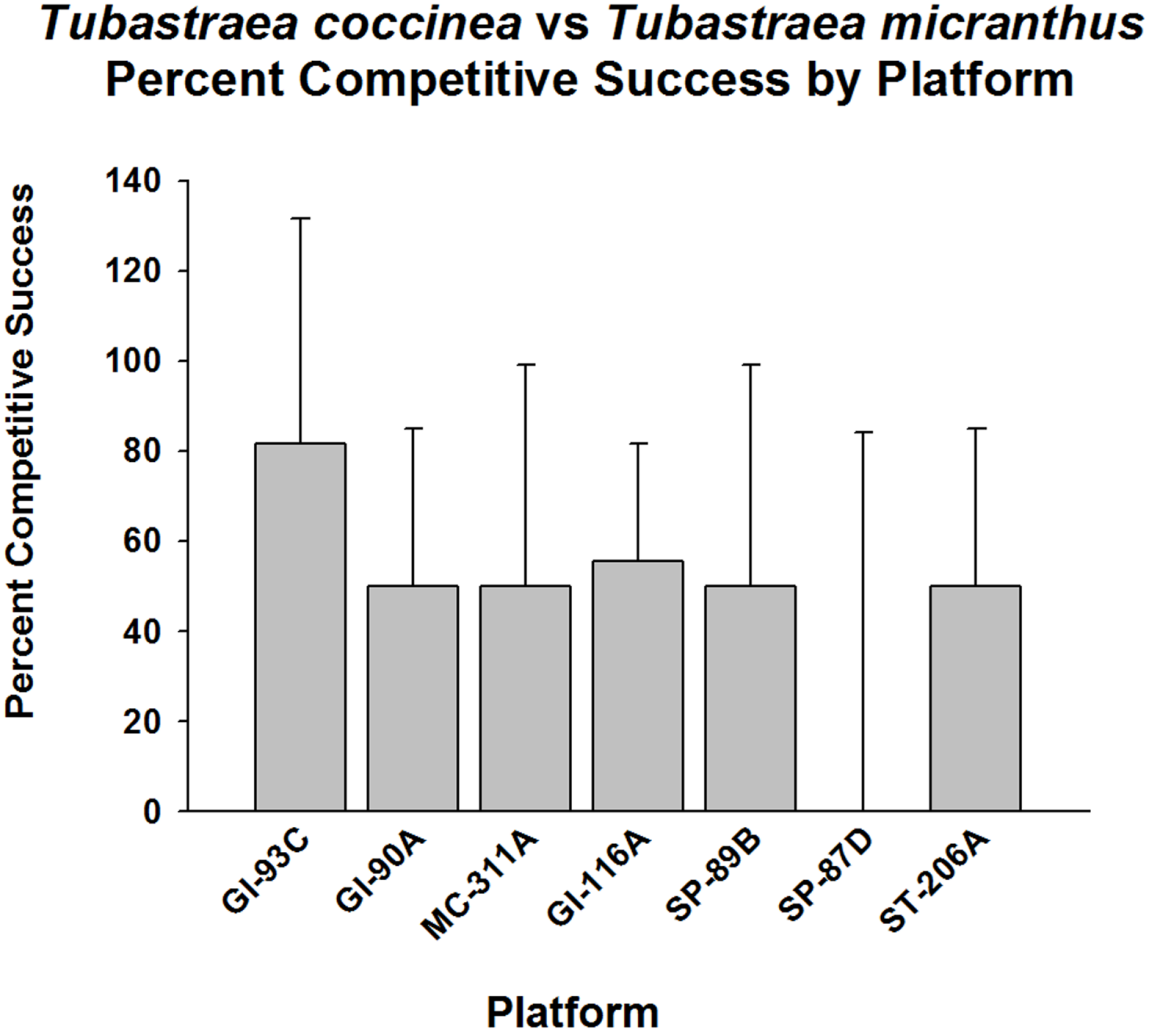
Percent of successes in competition for space between *Tubastraea coccinea* vs. *T*. *micranthus*. Here, *T*. *coccinea* was the target organism. Data presented by platform. Mean plus 95% confidence limits shown. Range of number of interactions per platform (n): 6–155. See Fig 2 legend for additional details. Overall competitive success significantly greater than 50% (p < 0.001, Fisher’s Exact Test). Significantly higher frequency of competitive successes on GI-93C than on other platforms (p < 0.001, Goodness of Fit Test, G-statistic) where competitive success frequency did not differ significantly from a 50% competitive success level (p>0.05

When frequency of competitive success was assessed between *Tubastraea coccinea* against *T*. *micranthus*, however, on average, *T*. *coccinea* was able to compete successfully with its congener for space more than 50% of the time (p < 0.001, Fisher’s Exact Test, Fig 5), varying between platforms. There was a higher frequency of competitive successes on GI-93C than on other platforms (p < 0.001, Goodness of Fit Test, G-statistic), where such did not vary from a 50% success level (p>0.05).

**Fig 6:**
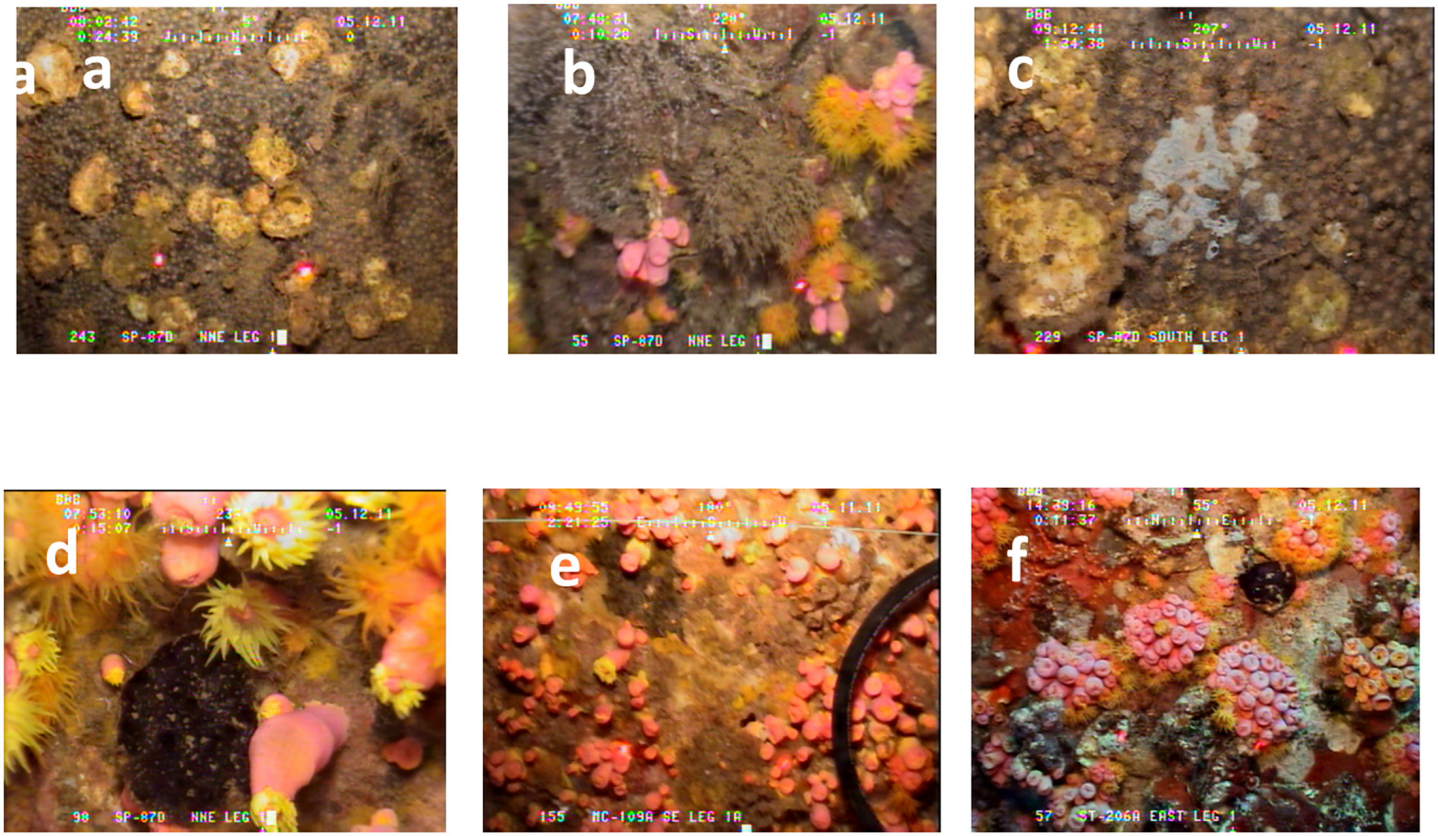
Photographs of the six most abundant competitors of space against *Tubastraea coccinea* on oil/gas platforms in the study region, northern Gulf of Mexico. Photos are still-captures from an ROV video. a = *Xestospongia* sp. with commensal zoanthids *Parazoanthus catenularis*; b = *Dictyonella funicularis*; c = *Haliclona vansoesti*; d = *Xestospongia carbonaria*; e = *Mycale carmigropila*; f = *Phorbas amaranthus*.

**Fig 7:**
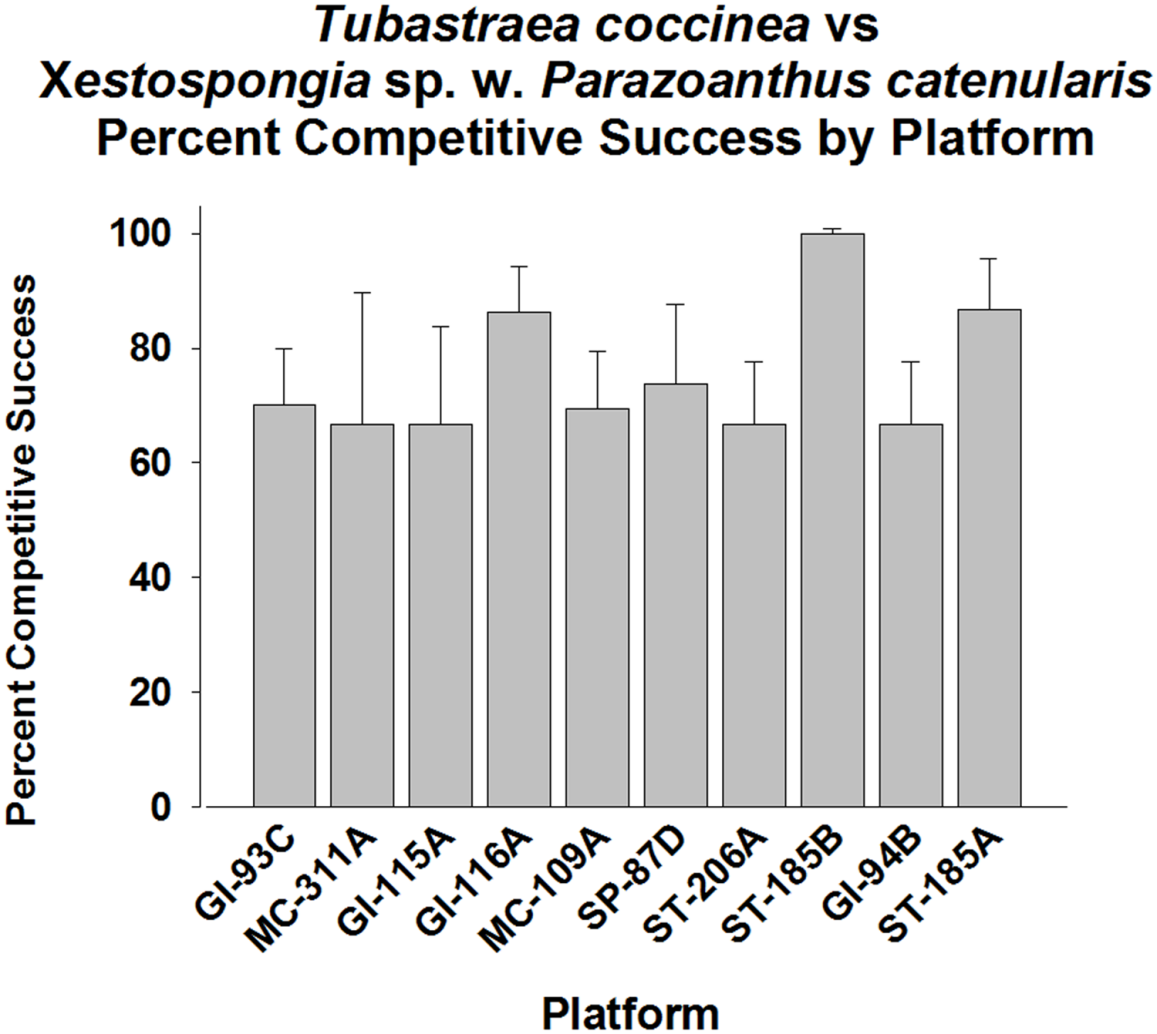
Percent of success in competition for space between *Tubastraea coccinea* vs. *Xestospongia* sp. (with commensal zoanthids *Parazoanthus catenularis*), by platform. Mean plus 95% confidence limits shown. Range of number of interactions per platform (n): 19–74. See Fig 2 legend for additional details. Overall competitive success significantly higher than the expected 50% (p < 0.001, Fisher’s Exact Test). Also, almost all platforms exhibited significantly high frequencies of competitive success (p < 0.05–0.001, Goodness of Fit Test, G-statistic). Only coral populations on MC-311A and GI-115A exhibited non-significant levels of competitive success (< 50% frequency of success, p > 0.05).

Since *Tubastraea coccinea* was more abundant and widely distributed on the platforms than *T*. *micranthus* [17,18], this facilitated assessment of *T*. *coccinea*’s competitive success against various epibenthic organisms. Here, only the most abundant associated species were assessed for frequency of competitive success. Six taxa emerged as common competitors of *T*. *coccinea*, all of them sponges (Fig 6, S1 Table). The first sponge was *Xestospongia* sp. (brown encrusting sponge bearing commensal zoanthids—*Parazoanthus catenularis*). *T*. *coccinea* was competitively superior to this sponge on all platforms, with an overall success rate of ~75%, ranging from 68–100% (p < 0.001, Fisher’s Exact Test, Fig 7). Almost all platforms exhibited high frequencies of competitive success (p < 0.05–0.001, Goodness of Fit Test, G-statistic), with only MC-311A and GI-115A not exhibiting levels different from 50% success (p > 0.05).

**Fig 8:**
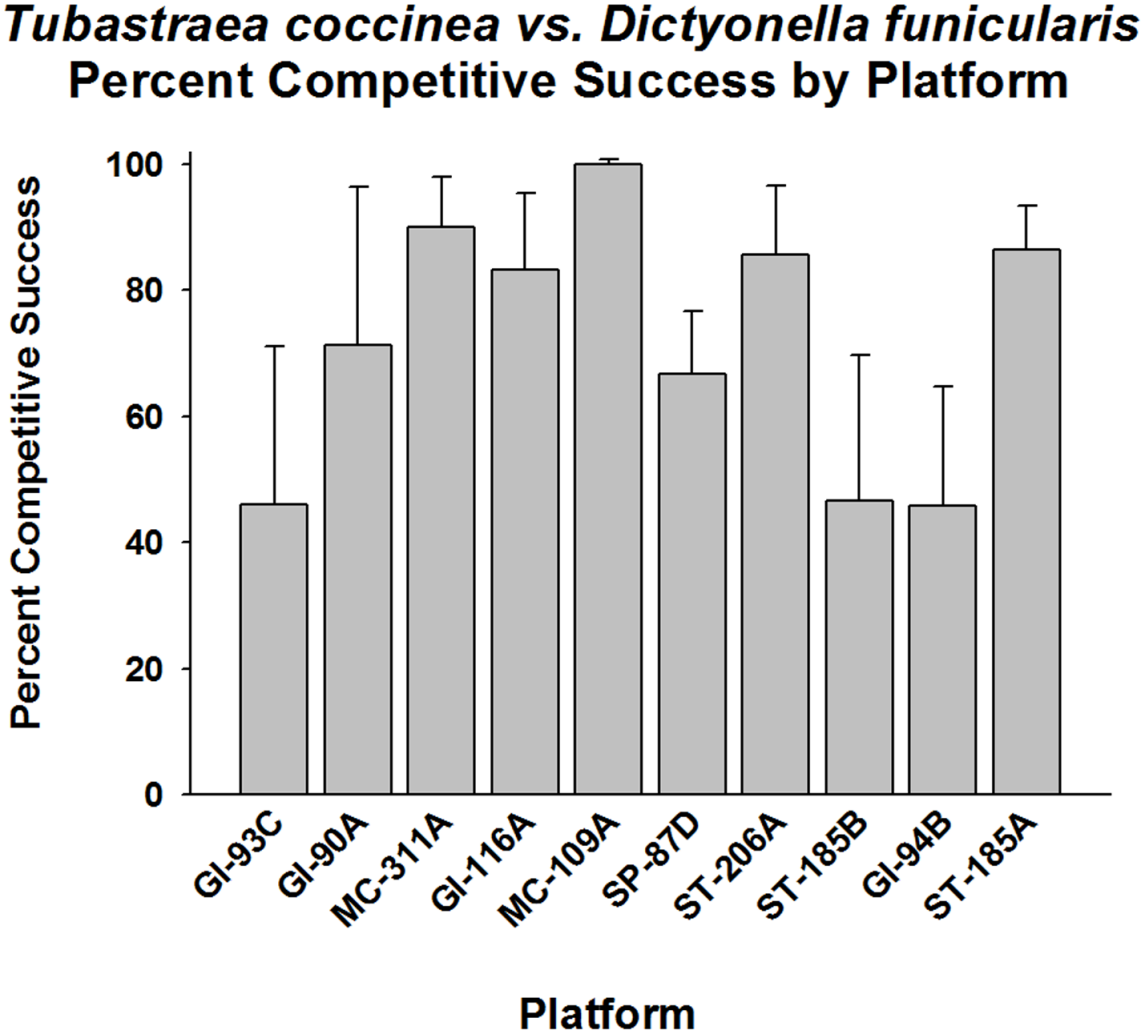
Percent of competitive success in competition for space between *Tubastraea coccinea* and *Dictyonella funicularis*, by platform. Mean plus 95% confidence limits shown. Range of number of interactions per platform (n): 13–83. See Fig 2 legend for additional details. Overall competitive successes significantly higher than 50% (p < 0.001, Fisher’s Exact Test). Highly variable win frequencies between platforms. Platforms exhibiting significant competitive success were GI-90A, MC-311A, GI-116A, MC-109A, SP-87D, and ST-185A (p < 0.01–0.001, Goodness of Fit Test, G-statistic). The remainder exhibited no significant difference with a 50% competitive success level (p > 0.05).

*Dictyonella finicularis* (light grey sponge) was also an abundant competitor for space with *T*. *coccinea*. The coral was competitively superior to this sponge overall (p < 0.001, Fisher’s Exact), but competitive success did vary among platforms (p < 0.01–0.001, Goodness of Fit Test, G-statistic). Competitive success and lack of such was equally distributed between platforms. That is, the platforms exhibiting competitive success were GI-90A, MC-311A, GI-116A, MC-109A, SP-87D, and ST-185A; the remainder did not exhibit any difference from a 50% competitive success level (p > 0.05). Competitive success frequencies varied between 45% and 100% between platform populations (Fig 8).

**Fig 9:**
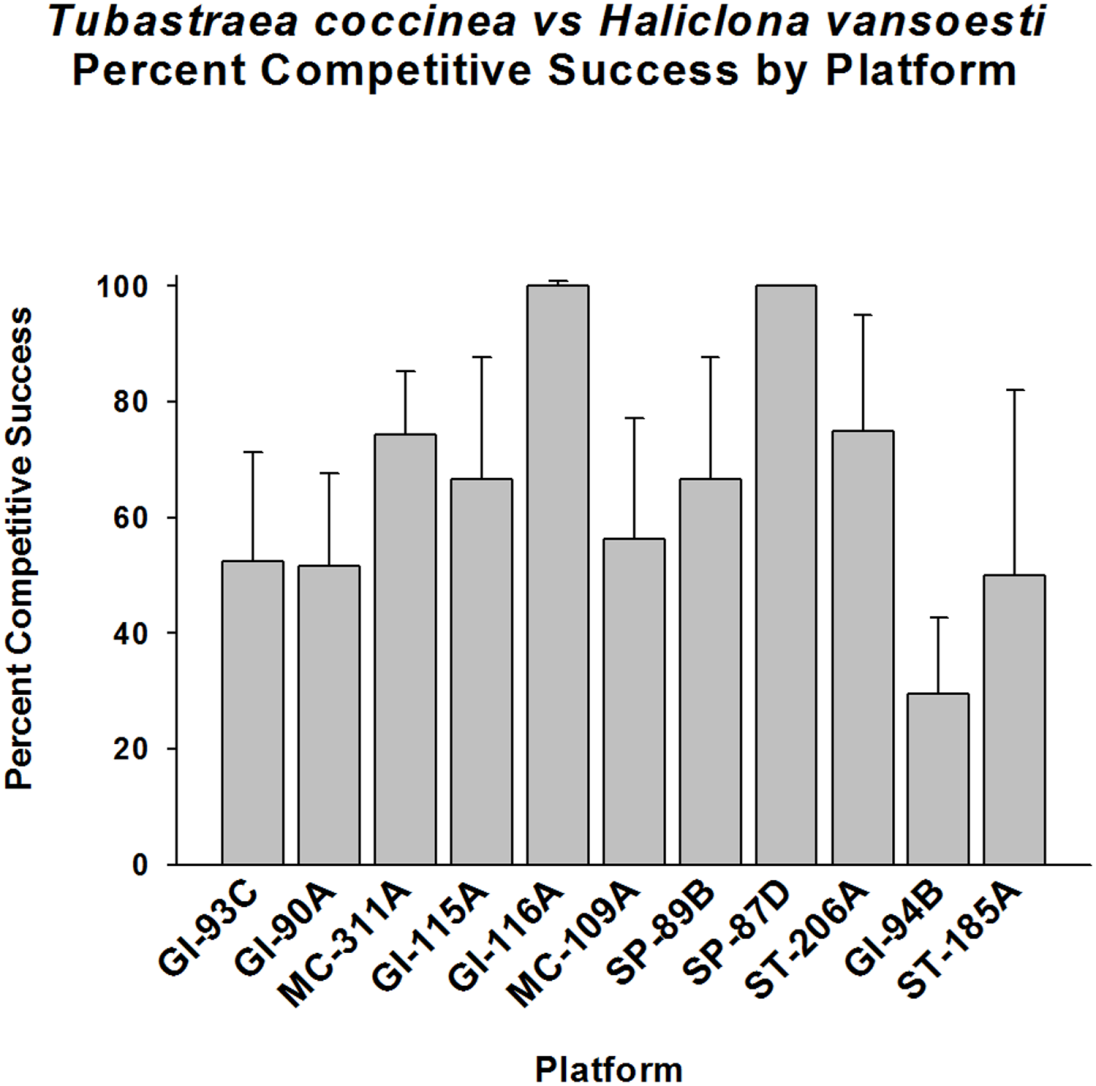
Percent of successes in competition for space between *Tubastraea coccinea* and *Haliclona vansoesti*, by platform. Mean plus 95% confidence limits shown. Range of number of interactions per platform (n): 16–60. See Fig 2 legend for additional details. No significant difference between overall observed frequency of competitive success, and an expected 1:1 ratio of the two competitors (p > 0.05, Fisher’s Exact Test). Highly variable success frequencies between platforms. *T*. *coccinea* populations on MC-311A, GI-115A, GI-116A, ST-185A, SP-89B, SP-87D, and ST-206A exhibited significant competitive success over this sponge (p < 0.05–0.01, Goodness of Fit Test, G-statistic). The GI-94B coral population exhibited significant competitive losses (p < 0.01). GI-93C, GI-90A, and MC-109A showed no significant variance from the expected 50% competitive success level (p >0.05).

Even greater variability was observed in competition between *Tubastraea coccinea* and *Haliclona vansoesti* (white encrusting sponge), also common on these platforms. On average, *T*. *coccinea* did not have any competitive edge over this organism, with a mean success rate of 60% (p > 0.05, Fisher’s Exact Test, Fig 9). The level of competitive success varied between platforms, however, ranging from 30% to 100% with high levels of competitive success on some platforms, and high variances between platforms. Competitive success and lack of such were distributed approximately equally between platforms. *T*. *coccinea* was competitively successful over this sponge on MC-311A, GI-115A, GI-116A, ST-185A, SP-89B, SP-87D, and ST-206A (p < 0.05–0.01, Goodness of Fit Tests, G-statistic). This coral exhibited losses on GI-94B, however, and did not show anyvariance from the expected 50% success level on GI-93C, GI-90A, and MC-109A (p >0.05).

**Fig 10:**
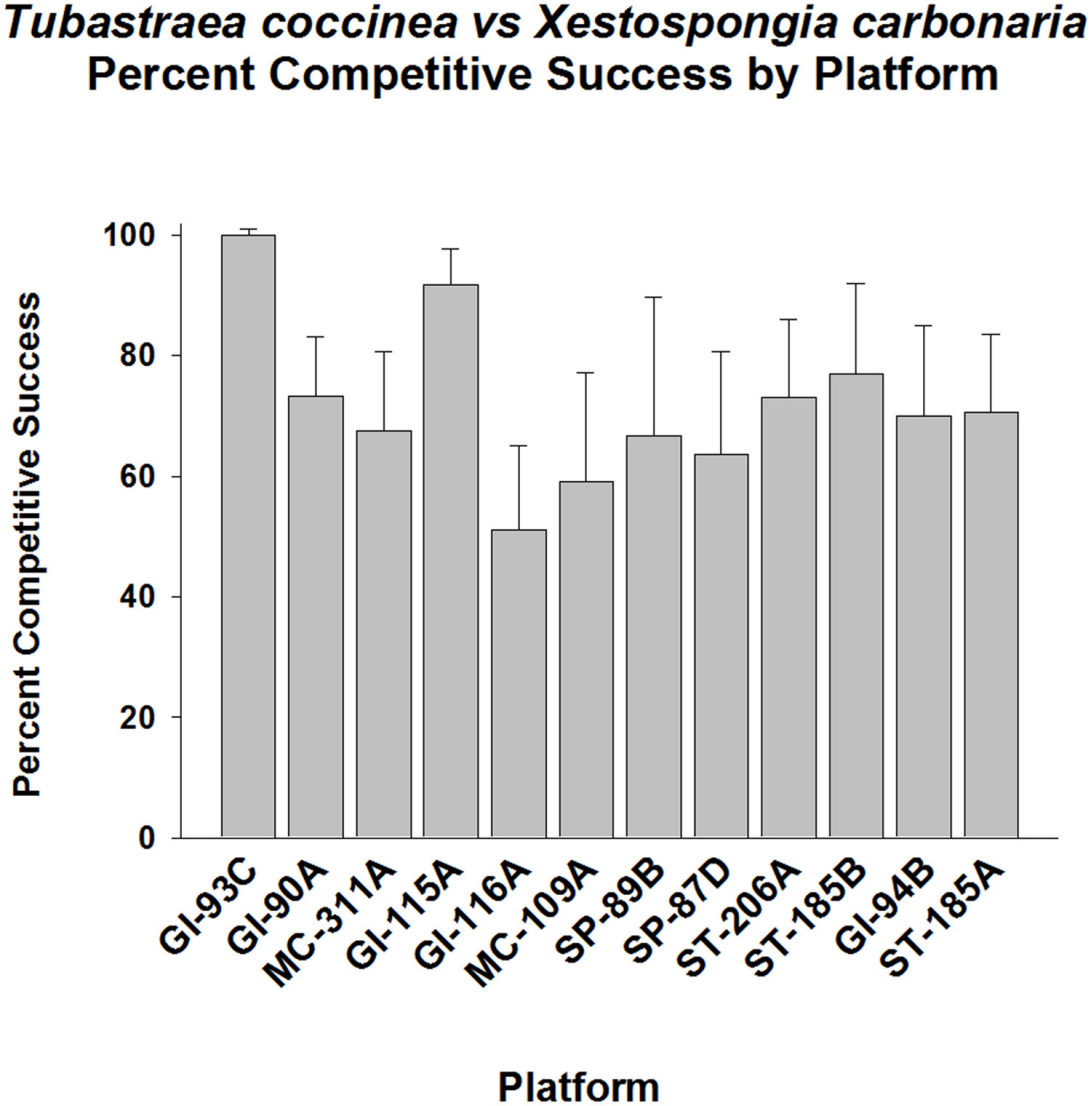
Percent of success in competition for space between *Tubastraea coccinea* and *Xestospongia carbonaria*, by platform. Mean plus 95% confidence limits shown. Range of number of interactions per platform (n): 20–58. See Fig 2 legend for additional details. Overall competitive success significantly higher than 50% (p < 0.001, Fisher’s Exact Test). Highly variable success frequencies between platforms. *T*. *coccinea* on GI-93C, GI-90A, MC-311A, GI-115A, GI-116A, ST-206A, and ST-185B all exhibited significantly high competitive success (p < 0.01–001, Goodness of Fit Test, G-statistic). The remainder exhibited competitive success frequencies which did notvary significantly from 50% (p> 0.05).

Another abundant competitor for space was *Xestopongia carbonaria* (common black encrusting sponge), which occurred in numerous small colonies at all depths. *Tubastraea coccinea* was a formidable competitor for space against this organism (p < 0.001, Fisher’s Exact Test), averaging ~70% wins (Fig 10). This competitive advantage varied from 50–100% between platforms. Despite these generally high levels of competitive success, this *T*. *coccinea* exhibited highly variable success frequencies between platforms. Once again, competitive success vs. lack thereof were approximately equally distributed among platform populations. *T*. *coccinea* was exhibited competitive success on GI-93C, GI-90A, MC-311A, GI-115A, GI-116A, ST-206A, and ST-185B (p < 0.01–001, Goodness of Fit Test, G-statistic). The remainder of platform populations did not exhibit success levels different than 50% (p> 0.05, Goodness of Fit Test, G-statistic).

**Fig 11:**
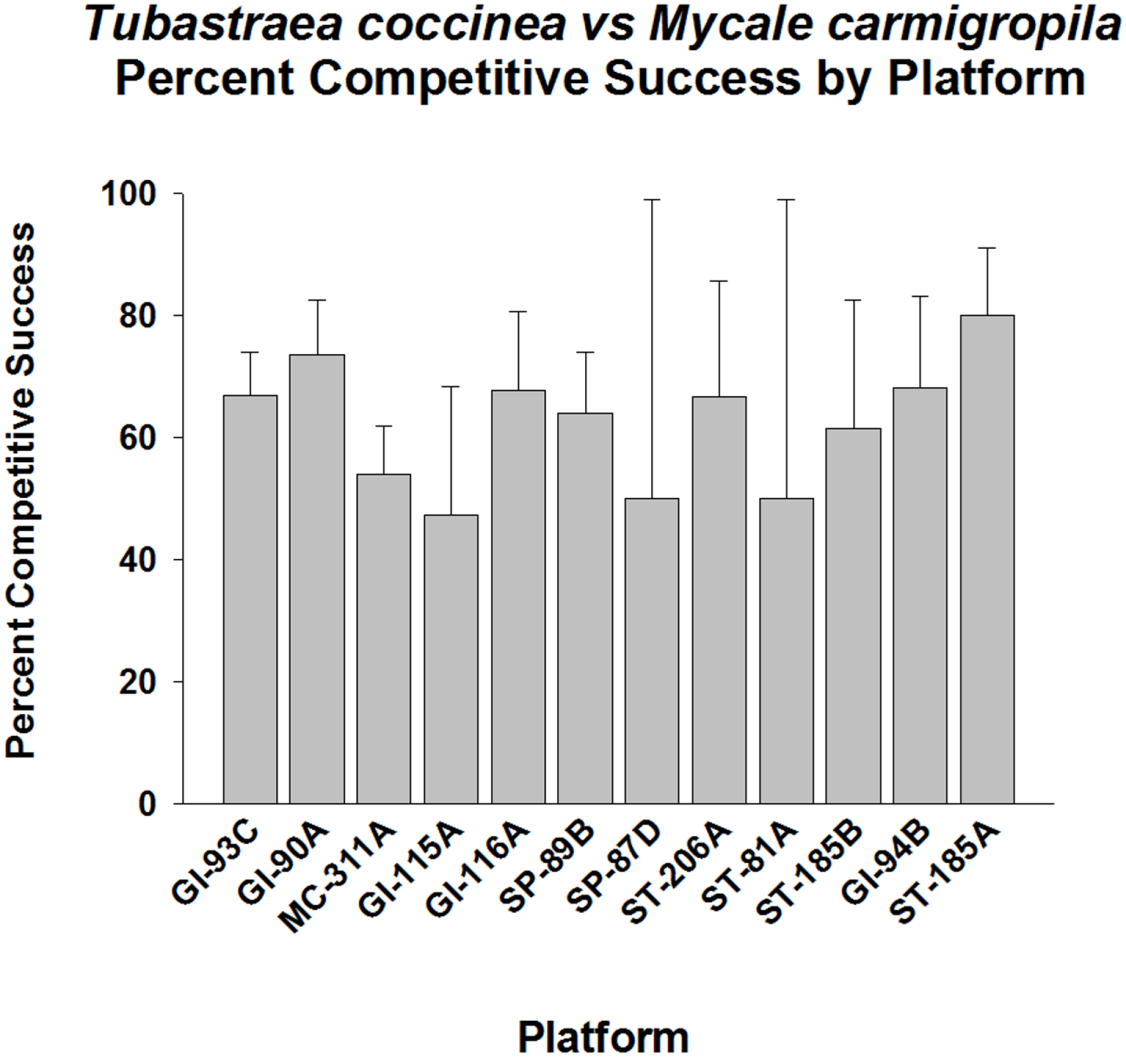
Percent success in competition for space between *Tubastraea coccinea* and *Mycale carmigropila*, by platform. Mean plus 95% confidence limits shown. Range of number of interactions per platform (n): 13–150. See Fig 2 legend for additional details. Overall competitive success significantly higher than 50% (p < 0.001, Fisher’s Exact Test, Goodness of Fit Test, G-statistic). *T*. *coccinea* populations were highly variable with respect to competitive success for this interaction. *T*. *coccinea* on GI-93C, GI-90A, GI-115A, GI-116A, SP-89B, and ST-185A all exhibited significantly high competitive success (p < 0.01–001, Goodness of Fit Test, G-statistic). Populations on the remainder of the platforms exhibited frequency of success not significantly different from 50% (p> 0.05).

*Mycale carmigropila* (brown encrusting sponge) was another abundant competitor for space with *Tubastraea coccinea*, and was generally larger in colony size than *Xestopongia carbonaria*. Again, *T*. *coccinea* was, on the average, competitively superior to this organism, at a frequency of 60% (p < 0.001, Fisher’s Exact Test, Goodness of Fit Test, G-statistic, Fig 11). Competitive success varied between platforms, ranging from 50–83%. *T*. *coccinea* exhibited high competitive success levels on GI-93C, GI-90A, GI-115A, GI-116A, SP-89B, and ST-185A (p < 0.01–001, Goodness of Fit Test, G-statistic). Populations on the remainder of the platforms did not exhibit competitive success frequencies greater than 50% in this interaction (p> 0.05).

**Fig 12:**
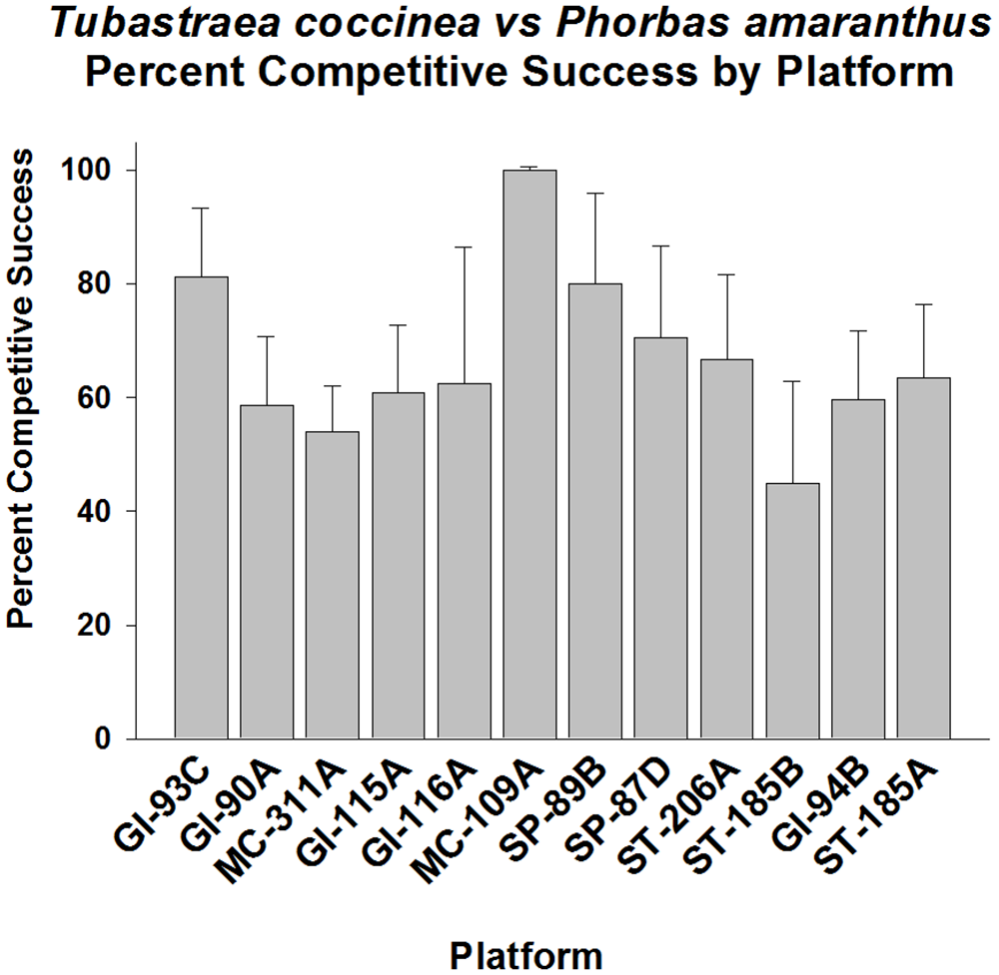
Percent of wins in competition for space between *Tubastraea coccinea* and *Phorbas amaranthus*, by platform. Mean plus 95% confidence limits shown. Range of number of interactions per platform (n): 17–150. See Fig 2 legend for additional details. Overall competitive success significantly higher than the 50% level (p < 0.01, Fisher’s Exact Test). Frequency of competitive success varied significantly between platforms. The following platforms exhibited significantly high competitive success frequencies: GI-93C, GI-90A, GI-116A, MC-109A, and SP89B (p < 0.05–0.01, Goodness of Fit Test, G-statistic). The remainder (most of the platforms) exhibited competitive success frequencies not significantly different from 50% (p > 0.05).

*Phorbas amaranthus* (common red encrusting sponge) was another primary competitor of *Tubastraea coccinea*. The coral was, on the average, successful in competition for space against this organism, again averaging about 60% competitive success (p < 0.01, Fisher’s Exact Test, Fig 12). Success varied between platforms, ranging from 45–100%, being particularly strong on several platforms. *T*. *coccinea* exhibited high competitive success on GI-93C, GI-90A, GI-116A, MC-109A, and SP89B (p < 0.05–0.01, Goodness of Fit Test, G-statistic). The remainder (most of the platforms) did not exhibitcompetitive success frequencies different from 50% (p > 0.05).

## Discussion

Our results reveal that both *Tubastraea micranthus*, the recent invasive species, and *T*. *coccinea*, an invasive that has been present for decades, are, on average, better competitors for space than the most abundant organisms they encountered in this study on these offshore platforms. There are two major implications that emerge from this study with respect to competitive success in *Tubastraea micranthus*. The first is that this species appears to have the capability to overgrow its sessile epibenthic neighbors [28,70] with an overall average competitive success frequency that did not fall below 50%.

Secondly, this competitive success is variable between platforms. It is possible that this is due to variation in environmental conditions experienced by each of the platforms. The study area is bounded in the north by the mouth of the Mississippi River and in the south by the blue-water conditions (the Mississippi Canyon). The discharge of the Mississippi is known to meander in all directions, including over the study area, carrying with it variable conditions of temperature, salinity, oxygen, nutrient concentrations, turbidity, pH, etc., causing the environmental conditions of the study sites to vary both latitudinally and longitudinally with time. Any of these variables could potentially affect outcome of competitive interactions.

We assume that the competitive outcomes observed in this study are due to the differential competitive abilities of the organisms involved in each individual encounter. It is possible that some of the interactions have been mediated by the environment, as is known to occur and has been demonstrated experimentally to occur between scleractinian corals and alcyonacean soft corals on the Great Barrier Reef [71]. In the case here in the Gulf of Mexico, the environments associated with different depths might have induced shifts in competitive advantage in *Tubastraea coccinea* and *T*. *micranthus* or their competitors. In a sister study, we recently suggested that *T*. *micranthus* may not grow as well as *T*. *coccinea* under conditions of high turbidity and high nutrients [8]. In the case of *T*. *coccinea*, however, the consistency of its competitive advantage suggests that there has been adaptation to its environment, species comprising the benthic community, and interactions within that community.

At present, there are no data to suggest that *T*. *micranthus* has successfully colonized any natural reef substratum in this region or elsewhere in the Gulf of Mexico. Thus, it is not yet known whether *T*. *micranthus* will possess the same competitive advantage it demonstrated here in a natural habitat, when colonizing different epibenthic communities or environments with different salinities, turbidities, sedimentation rates, nutrient regimes, etc. One thing is important, however. *T*. *coccinea* can monopolize artificial substrates but has proven not to be a threat to natural reef environments, such as coral reefs. There, in its new environment, it reverts to its natural habit of occurring in low densities and being cryptic, living beneath overhangs or in caves. The natural habit for *T*. *micranthus*, however, is exposed on the reef top. At this point, we do not know whether it will mimic the settlement and survival behavior of its congener or not.

The fact that comparative competitive success in these two congeners–*Tubastraea micranthus* and *T*. *coccinea*–was almost exactly the same when they were in competition with each other is an indicator that *T*. *micranthus*—the newer invasive species—may be an equally good competitor for space as its predecessor. Rejmanek [71] and Diez et al. [72] propose that when a second invasive species is a congener of the first one in an ecosystem, the probability of survival of the second invasive is dependent upon niche specificity and comparative competitive abilities. This is due to the possibility of competitive exclusion of the second invasive. Thus far, it appears that these two coral species are similar in their competitive abilities in their interactions with the native epifauna and also perceive each other immunologically as being “self”, not triggering aggressive interactions between them. They appear to be able to co-exist.

When one considers *Tubastraea coccinea* and its competitive success against all sessile epibiota pooled on these platforms, some differences become apparent in comparing similar competitive interactions involving *T*. *micranthus*. The first concerns the average frequency of competitive success in *T*. *coccinea*—which is 55%—significantly positive. This indicates that this species, despite having invaded the Gulf of Mexico several decades ago, is still an important competitor and is capable of maintaining its space and expanding its populations there. It has been integrated into the community and appears to coexist with its neighbors in a near-equilibrium situation. The second point is the consistency of the competitive success frequency in *T*. *coccinea*. There is very low variability in this frequency between platforms. This supports the suggestion that these older coral populations may be adapted to this new community and may have reached some level of equilibrium with respect to competitive efficacy in these communities.

*Tubastraea coccinea* has clearly been successful at procuring space for growth and reproduction in the western Atlantic, as is evident from its current geographic distribution, ranging from Brazil to the Florida Keys and the Gulf of Mexico [12,13,14,15,16,17,18,19]. Quantitative data regarding this species’ abilities in competition for space with the taxonomic groups it encountered is clear. They were strong with respect to *Xestospongia* sp, *Dictyonella funicularis*, and *Haliclona vansoesti*, all of which were encountered frequently. The low frequency of competitive success in these three sponges indicates that, in time, they may become less well represented in the affected benthic communities, at least on these artificial reefs. The high variability in the observed responses by these coral populations indicates that these competitive interactions may not yet have come to equilibrium. In addition, environmental variability has been demonstrated experimentally in the field to influence probability of competitive success [73]. In our case, *Dictyonella funicularis* appears to have a higher probability of out-competing this invasive coral under conditions of high turbidity and nutrient concentrations. The opposite was true for *H*. *vansoesti*, however, which exhibited a lower competitive advantage under those conditions. Laboratory experiments have shown that competitive abilities in *T*. *coccinea* vary species-specifically with the competitor [65]. We made similar observations here; that is, *T*. *coccinea*’s competitive abilities were found to be slightly higher than those of *Xestospongia carbonaria*, *Mycale carmigropila*, and *Phorbas amaranthus* than in other species.

The scleractinian coral *Tubastraea micranthus* appears to have the potential of being a strong new invasive species in the northern Gulf of Mexico, as its predecessor, *T*. *coccinea*, was and continues to be. *T*. *micranthus* populations have now expanded to 9 platforms in the Mississippi River region in over the period of at least a decade [8,21,22], as revealed by extensive ROV surveys throughout that region. *T*. *micranthus* appears to have similar competitive abilities to *T*. *coccinea* and may even be a stronger competitor for space. Its advantage in this new environment is enhanced by its preference to live at deeper depths—down to ≤ 138 m [22]. With the above documentation of *T*. *micranthus’* competitive abilities against the epifauna encountered in this study, it may have abilities comparable to *T*. *coccinea*, and could potentially be another successful invasive species in the western Atlantic. The competitive abilities of *T*. *micranthus* do not, of course, guarantee its success as an invasive in the tropical and sub-tropical western Atlantic. Successful competition for space is only one component necessary for its success. As mentioned earlier, factors such as tolerances to temperature and salinity [34], nutrient concentrations [35,36], turbidity, sedimentation, light [37], native diseases, lack of recruitment success, etc. are also important. Nonetheless, we now have a better understanding of one of these factors.

## Supporting Information

S1 Table Raw data, species competition matrix for *Tubastraea micranthus* and *T*. *coccinea*, competition results, by species. (XLS)

S2 Table Summary data, *Tubastraea micranthus* and *T*. *coccinea*, competition data, results by platform. (XLS)
